# Nasopharyngeal Vascular Hamartoma in a Dog

**DOI:** 10.1155/2020/9716179

**Published:** 2020-06-04

**Authors:** Annalisa N. Judy, Alexander I. Krebs, Joseph Haynes, Nina R. Kieves

**Affiliations:** ^1^The Ohio State University, Columbus OH, USA; ^2^Iowa State University, Ames, IA, USA

## Abstract

An 8-year-old spayed female 32 kg Labrador retriever was presented for further investigation into the underlying cause of dyspnea, stertor, and sleep apnea present for three months and worsening over 30 days. There were significant reduction in airflow through the nares and loud inspiratory stridor. Thoracic and cervical radiographs made were normal. A skull CT and retrograde rhinoscopy showed a mass occluding the majority of the nasopharynx above the caudal third of the hard palate. The main differential diagnoses included a neoplastic mass vs. inflammatory mass vs. cyst vs. mucous obstruction. There was no destruction of nasal turbinates, making a benign etiology more likely. Biopsy of the mass showed an inflammatory process. En bloc excision of the mass was performed via ventral rhinotomy without complication. Histopathology of the excised mass revealed it to be a mucosal vascular hamartoma. The dog recovered uneventfully and had no further respiratory issues, short or long term. Although vascular hamartomas are a rare finding in veterinary medicine, they can be found in a wide variety of species and anatomic locations. They should be considered when naming differentials for benign mass lesions throughout the body, including the nasopharynx. Although they are benign masses in nature, they can be clinically significant and should be addressed. Prognosis after removal in this location is excellent.

## 1. Introduction

Hamartomas are masses that consist of excessive growth of mature and normal tissues found in the area where the hamartoma is forming. Specifically, a vascular hamartoma is a benign and disorganized growth of vasculature tissue [1, 2]. The benign nature of these tumors and their limited ability to grow indicate that these are more developmental lesions rather than true neoplasms, though they commonly resemble a neoplastic growth on gross examination. Vascular hamartomas are a rare condition reported in dogs, cats, horses, and cows [3]. Hamartomas have been reported in the brain, spinal cord, nasal cavity, mandible, viscera, and musculature in these species. Although these lesions do not have the capability of metastasis and have a limited ability to grow, they can be clinically significant and should be considered a differential diagnosis in veterinary medicine. It has been reported that vascular hamartomas can rupture and cause hemorrhage spontaneously, while large hamartomas can cause a mass effect and compression/occlusion if growing in a confined area [3].

## 2. Case Description

An 8-year-old spayed female Labrador retriever weighing 32.3 kg (71 lbs) was presented to the Iowa State University Lloyd Small Animal Hospital's Internal Medicine Service for evaluation of chronic snoring and wheezing. The dog had noticeable difficulty in breathing for three-month duration with progressing severity 30 days prior to presentation. The client noticed that the dog had been pawing at her nose and rubbing it on the ground throughout the day with increasing frequency. There were no reports of fainting or loss of consciousness at home, but sleep apnea had been reported. It was reported that the dog would frequently wake up gasping after prolonged periods of attempting to breathe through its nose. The clients believed that the dog generally seemed most comfortable while panting. They also reported that the dog had difficulty in eating due to its inability to breathe through its nose, although it continued to have a normal appetite and interest in food. An initial workup was performed by the primary care veterinarian, which included a nasal endoscopy. The procedure was unable to visualize the entire nasal cavity and was inconclusive. Therefore, the primary care veterinarian believed laryngeal paralysis to be the primary differential diagnosis. Medical treatment of the clinical signs had not been attempted, and the dog was referred to Iowa State University at that time.

On presentation to Iowa State University, the dog appeared bright, alert, and responsive. Air movement through the nares was absent bilaterally, and there was mucopurulent discharge from the right naris. No facial deformation or asymmetry was present, and no pain was elicited with facial palpation. There was no resistance or pain with ocular retropulsion of either eye, and vision was intact. Respiratory noise was quite audible, sounding like a snore during inspiration. During thoracic auscultation, the heart and lungs sounded normal, but there was pronounced referred upper respiratory noise. The remainder of the physical examination had no additional significant findings or abnormalities.

**Figure 1:**
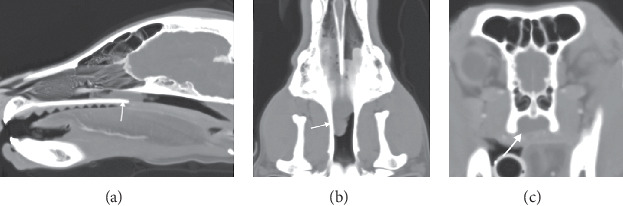
Sagittal (a), dorsal (b), and axial (c) CT images in soft tissue window illustrating a soft tissue/fluid dense material (white arrows) in both ventral nasal passages, slightly more in the right side, beginning at the second maxillary premolars. At the caudal third of the hard palate, the nasopharynx becomes completely occluded by the mass structure (best seen on the axial view (c)). No destruction of the ethmoturbinates is identified, and the cribriform plate is intact. The sinuses are unaffected.

A complete blood count, chemistry panel, and coagulation panel were run and were unremarkable besides a mildly elevated alkaline phosphatase of 257 IU/L (reference range = 20-150 IU/L). Thoracic and cervical radiographs were made and showed no abnormalities. Based on the upper respiratory signs and the bilateral lack of airflow through the nares, a sedated oral examination and skull CT were performed. The larynx was visualized under sedation and appeared to be functionally normal, ruling out laryngeal paralysis as a cause of the dog's clinical signs. Skull CT showed a soft tissue/fluid-attenuated structure in the nasopharynx at the junction of the soft and hard palate. The mass-like structure appeared to occlude the majority of the nasopharynx and was more present on the right side than on the left. There was no turbinate destruction or boney lysis appreciated on the CT (Figure 1).

Based on the CT and physical exam findings, a mass lesion was suspected. Specific differentials included a cystic structure, neoplastic mass (malignant or benign), inflammatory mass, or a mucoid obstruction. Specific neoplasms considered included respiratory epithelial carcinoma, nasal adenocarcinoma, chondrosarcoma, and squamous cell carcinoma based on literature and the frequency of neoplasms of those etiologies occurring in the nasopharyngeal location [4].

After the mass-like structure was seen on a CT scan, a retrograde rhinoscopy was performed to obtain biopsies of the mass. Upon rhinoscopy, the mass was clearly visualized and noted to be a tubular-like structure that was red-yellow in color. It was first seen in the caudal nasopharynx and extended rostrally, although the rostral border could not be appreciated. The mucosa and turbinates surrounding the mass looked healthy and had no gross evidence of metastasis or destruction. The mass was biopsied and showed chronic, severe lymphoplasmacytic nasopharyngitis. It was also noted that there was a significant amount of hemorrhage after these biopsies were taken. The nasal mucosa was also biopsied and revealed a diffuse subacute to chronic edematous and lymphoplasmacytic rhinitis with purulent and ulcerative rhinitis. The regional lymph nodes were aspirated and revealed moderate eosinophilic and mild neutrophilic inflammation. Overall, based on histopathology, the mass appeared to be inflammatory and benign in etiology, but no definitive diagnosis was made.

The lack of nasal turbinate destruction and biopsy results made a malignant neoplastic differential less likely than a benign or inflammatory mass. The lack of local and distant metastasis indicated that surgical extraction was a good option for the treatment of the dog's clinical condition, and surgical excision was pursued. A ventral rhinotomy approach was used because of the decreased likelihood of intraoperative complications compared to a dorsal rhinotomy [5] and better visualization given the caudal location of the mass [6].

**Figure 2:**
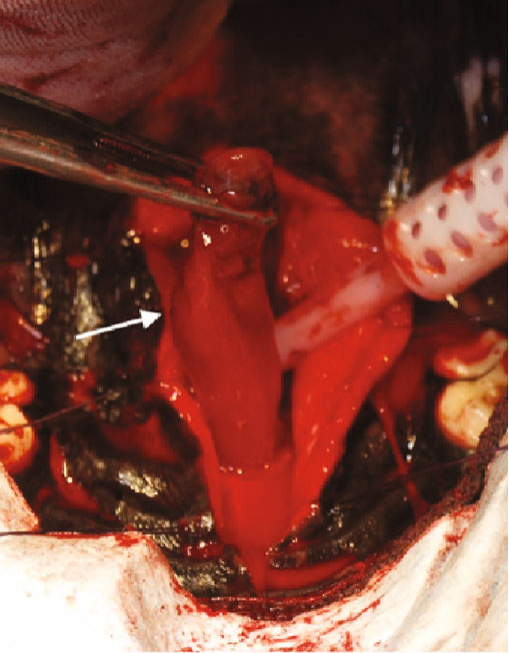
Intraoperative imaging showing the mass (white arrow) being elevated from the nasopharynx via ventral rhinotomy prior to en bloc resection at its stalk. The dog is in dorsal recumbency with rostral being the bottom of the photo.

The dog was placed under general anesthesia for mass removal via a ventral rhinotomy. A midline incision was made down the center of the soft palate extending caudally from the junction of the hard palate and soft palate. The incision was extended cranially through the mucosa of the hard palate to the palatine bone, and a stab incision was made through the nasal mucosa at the juncture of the soft and hard palate. Once the ventral aspect of the nasopharynx was opened, the mass was easily visualized within the nasopharynx. The mass was more present on the right side of the nasal cavity than on the left and had a red to yellow color. The cranial aspect of the mass was attached to the mucosa, while the caudal aspect was freely movable. Hemostasis of the mucosa was achieved through digital pressure rather than electrocautery to improve postoperative healing. The mucoperiosteum of the hard palate was elevated laterally to the alveolar ridge. The mucoperiosteum and soft palate attachments to the caudal edge of the palatine bone were incised. The edges of the incision were retracted using stay sutures on each side. A 1 cm × 3 mm block of palatine bone was removed starting at the junction of the hard and soft palate. Portions of the bone were submitted for histopathology. The approximately 4 cm × 2 cm mass had a stalk that extended dorsally and rostrally that appeared to be attached to the nasal mucosa (Figure 2). This stalk was elevated from the mucosa, and the mass was removed en bloc via sharp dissection in an attempt to obtain clean margins. The mass was submitted for histopathology en bloc. Small pieces (approximately 2 mm in size) of nasopharyngeal mucosa were also submitted for histopathology, and a culture was taken of the nasopharyngeal area. The area was lavaged with sterile saline following removal of the mass. A red rubber catheter was passed through the right ventral nasal meatus and flushed with sterile saline. This was repeated on the left side. The stay sutures were then removed, and the incision was closed routinely in multiple layers. The dog recovered uneventfully from anesthesia and was monitored in the ICU after surgery.

Postoperatively, the dog experienced no significant complications in the ICU. The dog seemed immediately more comfortable breathing through its nose after surgery. The dog was maintained on IV fluids and pain relief medications for 24 hours postoperatively and was monitored for any signs of hemorrhage. She was discharged the day following surgery on oral carprofen (Rimadyl, Zoetis Petcare, Parsippany-Troy Hills, New Jersey) 2.2 mg/kg (1 mg/lb) every 12 hours and oral tramadol hydrochloride (Ultram, Sun Pharmaceuticals Industries Ltd., Goregaon, Mumbai, India) 3 mg/kg (1.4 mg/lb) every 8-12 hours. The owners were instructed to feed a soft food diet and to not allow anything in the mouth such as chew toys for three weeks postoperatively to minimize trauma to the surgical site and area where part of the hard palate was removed.

**Figure 3:**
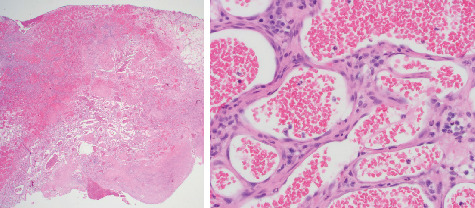
Ulcerated polypoid mass from nasal mucosa. The mass is composed of a fibrovascular core containing numerous blood-filled sinusoidal spaces, a few of which also contained a thrombus.

The polypoid mass was composed of a fibrovascular core covered with a stratified squamous epithelium that was ulcerated in several foci. The core contained numerous blood-filled sinusoidal structures, a few of which also contained a thrombus (Figure 3). In addition, there was a moderate diffuse infiltrate of lymphocytes, plasma cells, and scattered polymorphonuclear leukocytes throughout the core. The final histopathologic diagnosis was mucosal vascular hamartoma. Based on this benign result, no additional therapy was recommended.

Recheck exam 2 weeks postoperatively performed by the primary care veterinarian indicated that the surgical site was healing, and the owners reported a smooth recovery after surgery. No recurrence of respiratory issues had occurred since the surgery. Additionally, no abnormalities were noted on a physical exam performed 1 year following surgery in regard to recurrence of the mass or ongoing signs of nasopharyngeal occlusion and respiratory issues. The dog had maintained a good appetite, gained weight, and remained in good overall health. At the time of write-up, the dog is still alive and has had no recurrence of symptoms (7 years postsurgery).

## 3. Discussion

This case report discusses the diagnostic approach and treatment of a nasal mucosal vascular hamartoma in a dog, which has not been reported before in the veterinary literature. This case demonstrates how hamartomas can be clinically significant and should be considered as a differential for a mass-like lesion seen on imaging and causing clinical signs.

Hamartomas are a rare occurrence in veterinary medicine. They occur when there is excessive growth of normal mature tissue in a discrete area. Grossly, they are unable to be differentiated from neoplastic masses and require a histopathologic evaluation for a definitive diagnosis. Hamartomas have been described in numerous locations in many species including dogs and humans [3]. As hamartomas do not have the ability to metastasize, their clinical relevance is in local disruption of normal tissues. In this instance, the mass was large enough to occlude the majority of the nasopharynx of the dog, significantly inhibiting its ability to breath. In addition to becoming space-occupying masses, vascular hamartomas in particular have been reported to rupture and spontaneously hemorrhage [3].

A similar case of a hamartoma was reported in a 3-year-old female cat in Australia. The cat was presented for chronic right-sided epiphora, sneezing, and occasional protrusion of the mass out of the right naris. Biopsy of the mass and the lack of boney destruction on skull CT indicated that it was a benign inflammatory condition rather than neoplastic. The main differentials for this case were a nasopharyngeal inflammatory polyp, granulomatous disease, Cryptococcus, or a foreign body. Based on the benign etiology of the mass, surgical excision was elected as the treatment option. The nasopharynx was approached via ventral rhinotomy. Appropriate exposure was achieved, and the mass was removed from the nasopharynx. The remainder of the nasopharyngeal structures and mucosa appeared normal. The mass was submitted for histopathology and diagnosed as a vascular hamartoma. Postoperatively, the cat did well and had no complications recovering, except for some mild anorexia that resolved once the cat's normal diet was offered. Ten-month postoperative recheck revealed that the cat had healed well and had no further problems [7].

There are numerous approaches to the nasal cavity and nasopharynx described in both veterinary and human medicine. The ventral rhinotomy approach to removing nasopharyngeal masses appears to be the best choice, as it has superior visualization of the nasopharyngeal area, and patients seem to recover with minimal to no short- or long-term complications [5]. A dorsal rhinotomy approach has also been described in veterinary medicine and is most appropriately used when attempting to access any area of the nasal cavity except the nasopharynx, as it offers superior visualization of the nasal cavity and the frontal sinuses [5]. However, this approach can require that the common carotid artery be occluded, which can lead to increased intraoperative complications. Dorsal rhinotomy is also associated with more subcutaneous emphysema postoperatively, when compared to a ventral approach [5]. Other approaches to the nasal cavity and nasopharynx have been described in human medicine. A lateral rhinotomy approach provides superior visualization of the nasal cavity, and this approach has been used to successfully remove nasal angiofibromas [8]. Another approach is midfacial degloving, which allows for good visualization of the nasal cavity and has excellent cosmetic healing [9]. This approach has not been used in veterinary medicine due to the high level of dissection required and the decreased necessity for cosmesis in veterinary patients. In this specific case, a ventral rhinotomy approach was deemed most appropriate based on the location of the mass and the minimized complications associated with this approach compared to the others described. With superior visualization, the mass was removed en bloc, which was a curative treatment for this patient.

The feline case and the canine case discussed above both show similarities in diagnostic and treatment approach, as well as short-term and long-term recovery. These cases indicate that vascular hamartomas should be considered differential diagnoses when faced with an obstructive, clinically significant, nonaggressive mass lesion in the nasopharynx. The benign nature of hamartomas leads to a straightforward treatment plan and excellent prognosis compared to the more common neoplastic tumors found in the nasopharynx, with surgical excision being curative.
